# Chasing Migration Genes: A Brain Expressed Sequence Tag Resource for Summer and Migratory Monarch Butterflies (*Danaus plexippus*)

**DOI:** 10.1371/journal.pone.0001345

**Published:** 2008-01-09

**Authors:** Haisun Zhu, Amy Casselman, Steven M. Reppert

**Affiliations:** Department of Neurobiology, University of Massachusetts Medical School, Worcester, Massachusetts, United States of America; Minnesota State University Mankato, United States of America

## Abstract

North American monarch butterflies (*Danaus plexippus*) undergo a spectacular fall migration. In contrast to summer butterflies, migrants are juvenile hormone (JH) deficient, which leads to reproductive diapause and increased longevity. Migrants also utilize time-compensated sun compass orientation to help them navigate to their overwintering grounds. Here, we describe a brain expressed sequence tag (EST) resource to identify genes involved in migratory behaviors. A brain EST library was constructed from summer and migrating butterflies. Of 9,484 unique sequences, 6068 had positive hits with the non-redundant protein database; the EST database likely represents ∼52% of the gene-encoding potential of the monarch genome. The brain transcriptome was cataloged using Gene Ontology and compared to *Drosophila.* Monarch genes were well represented, including those implicated in behavior. Three genes involved in increased JH activity (*allatotropin*, *juvenile hormone acid methyltransfersase*, and *takeout*) were upregulated in summer butterflies, compared to migrants. The locomotion-relevant *turtle* gene was marginally upregulated in migrants, while the *foraging* and *single-minded* genes were not differentially regulated. Many of the genes important for the monarch circadian clock mechanism (involved in sun compass orientation) were in the EST resource, including the newly identified *cryptochrome 2*. The EST database also revealed a novel Na+/K+ ATPase allele predicted to be more resistant to the toxic effects of milkweed than that reported previously. Potential genetic markers were identified from 3,486 EST contigs and included 1599 double-hit single nucleotide polymorphisms (SNPs) and 98 microsatellite polymorphisms. These data provide a template of the brain transcriptome for the monarch butterfly. Our “snap-shot” analysis of the differential regulation of candidate genes between summer and migratory butterflies suggests that unbiased, comprehensive transcriptional profiling will inform the molecular basis of migration. The identified SNPs and microsatellite polymorphisms can be used as genetic markers to address questions of population and subspecies structure.

## Introduction

The monarch butterfly (*Danaus plexippus*) is arguably the world's most captivating and well-known butterfly species [1]. Monarchs are renowned for their orange and black-edged wings, milkweed-derived chemical defenses, and involvement in mimicry with viceroy butterflies. But the monarch's most notable claim to fame is the spectacular fall migration of its North American populations.

The migratory state is characterized by reproductive diapause, a condition in which the butterflies exhibit refractory mating behavior and arrested reproductive development, as migrants need to conserve energy for the long journey [2]. Migrants also have increased abdominal fat stores, a marked increase in longevity, and an overwhelming urge to fly south. Diapause persists in Eastern North American migrants at the overwintering sites in Mexico until the early spring when the butterflies reproduce and take wing northward to lay fertilized eggs on newly emerged milkweed plants (genus *Asclepias*) in the southern United States. Another two to three generations of reproductively competent, short-lived “summer” butterflies follow the progressive, northward emergence of milkweed to reestablish, by late summer, the most northerly reaches (in southern Canada) of the eastern population of monarch butterflies. In the fall, decreasing daylength helps trigger the migratory generation and, once again, the long journey south begins [2]–[4].

As in *Drosophila melanogaster*, juvenile hormone (JH) is a key regulator of adult reproductive activity and longevity in monarch butterflies [5]. In migratory monarchs, JH levels are significantly reduced, reproductive development is curtailed, and longevity is increased-from a life span of a few weeks in summer butterflies to several months in migrants. Moreover, experimental manipulation of JH in adult butterflies causes predictable changes in reproductive activity and longevity. Thus, reproductive diapause and increased longevity, phenotypic markers of the migratory state, are induced by JH deficiency. JH synthesis is likely regulated by insulin-like peptides originating from neurosecretory cells in the pars intercerebralis [6].

The circadian clock plays a vital role in monarch migration by providing the timing component of time-compensated sun compass orientation [7]–[10], which contributes to navigation to the overwintering grounds. The remarkable navigational abilities of monarch butterflies are part of a genetic program, as the migrants are always on their maiden voyage, and those that make the trip south are at least two generations removed from the previous generation of migrants [3]. Here, we describe an expressed sequence tag (EST) resource, as a tool for ultimately identifying genes involved in migratory behaviors, as well as in other aspects of the biology of monarch butterflies.

## Results and Discussion

### Monarch brain EST database

**Figure 1:**
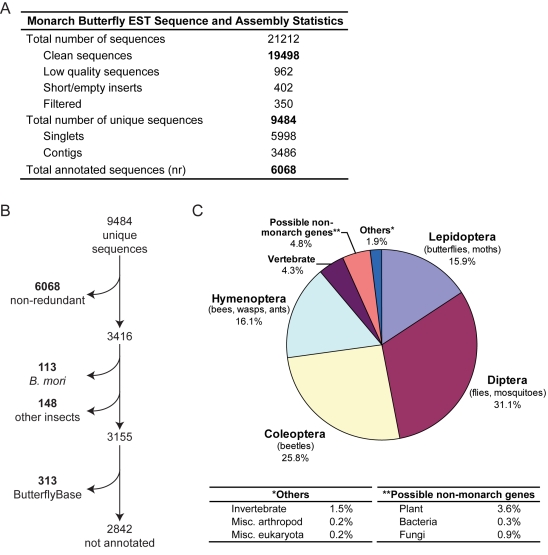
Overview of the monarch brain EST database. A. Sequencing the monarch brain cDNA clones and assembly into contigs. B. Annotating the monarch EST database (described in text). C. The 6068 ESTs annotated against the non-redundant protein database are represented in the pie-chart according to the best matching sequences.

Nearly 300 monarch brains were collected from a mix of summer reproductive animals and fall migrating animals (Table 1) to create a cDNA library (average insert size 1.7 kb). Library clones were sequenced at the 5′ ends to create the brain EST database. The average read length was 741 base pairs. Out of 21,212 sequence reads, 19,498 were classified as “clean” sequences (GenBank accession numbers EY255129–EY274705) (Dataset S1). These were assembled into 3,486 contigs and 5,998 singlets, resulting in a total of 9,484 unique sequences (Fig 1A). The monarch butterfly EST Information Management Application (ESTIMA) can be found at: http://titan.biotec.uiuc.edu/cgi-bin/ESTWebsite/estima_startseqSetbutterfly

**Table 1:** Monarch Butterflies Collected for the cDNA Library

State*	Capture date	#Males	#Females	Total
MA	August 11, 2004	19	17	36
MA	August 12, 2004	20	19	39
MA	August 14, 2004	20	21	41
MN	September 5, 2004	10	29	39
MN	September 6, 2004	20	21	41
MN	September 7, 2004	30	10	40
TX	October 19, 2004	20	20	40
TX	October 20, 2004	8	14	22
**Total**		147	151	298

* Within the United States of America. MA, Massachusetts; MN, Minnesota; TX, Texas.

### Database matches

The 9,484 unique sequences were compared to the non-redundant (nr) protein database (NCBI) using the BLASTX algorithm. Of these, 6068 matched an nr entry at E≤1×10^−5^ (Fig. 1B). Nearly 16% of these sequences had a best hit among the Lepidoptera, but surprisingly 31% had a best hit within the Diptera (Fig. 1C). This discrepancy is likely due to the fact that many dipteran genomes have been sequenced, and the only lepidopteran genome available is that of the commercial silkworm *Bombyx mori*. The annotation also revealed a small number of sequences that are similar to plants. These are mostly likely due to pollen contaminants in the brain dissections. In addition, a small number of bacterial and fungal genes were identified; these probably represent parasitic infections of some of our summer butterflies. Three sequences with similarity to *Nosema* species were discovered in the annotation. *Nosema* is known to be an infectious microsporidian in Lepidoptera [11].

Of the 3416 ESTs that did not have a match with the nr database, 113 had at least one match with the *B. mori* UniGene database (E≤1×10^−5^), and 148 had at least one hit with one or more of the following protein databases (NCBI): *Tribolium castaneum*, Flybase, *Apis mellifera*, and *Anopheles gambiae*. Of the remaining 3155 ESTs, 313 had a hit with the ButterflyBase v2.9 (Consortium for Comparative Genomics of Lepidoptera; http://heliconius.cap.ed.ac.uk/butterfly/db/index.php) (Fig. 1B). The ButterflyBase database used for the search includes EST sequences from 20 lepidopteran species, excluding *B. mori*. To determine if the remaining 2842 sequences with no matches have the potential to encode proteins, we used the OrfPredictor web server (https://fungalgenome.concordia.ca/tools/OrfPredictor.html; [12]). A total of 2563 ESTs (90%) were predicted to contain an ORF of 30 amino acids or longer, and 2473 (96%) of these were encoded on the plus strand, which is expected as the library was directionally cloned. Many of these non-annotated ESTs may represent genes unique to butterflies, the butterfly family Nymphalidae, and/or monarchs.

### Number of genes

Our EST database likely represents a large portion of the gene-encoding potential of the monarch genome. After the sequences similar to plant, bacterial, and fungal genes were removed from the unique sequences tally, 9024 monarch sequences still remain. The unique sequence tally may be a modest overestimate (∼20%) of the actual number of monarch genes in the database, however, because assembly into contigs is not perfect [13]. Using a conservative estimate of 7219 unique genes, our database could represent ∼39% of the monarch protein-coding sequences, compared to the *B. mori* genome (18,510 genes predicted; Table 2). Yet, the monarch butterfly has the smallest genome of the lepidopterans examined (based on 59 lepidopterans from Animal Genome Size Database http://www.genomesize.com [14]), and is more similar in size to that of the mosquito *A. gambiae*, which is predicted to contain 13,683 genes (Table 2). Therefore, compared to the mosquito genome, our EST database could represent ∼52% of the genes in the monarch genome. Furthermore, as our EST database is based on a brain library, it is likely that our EST database represents more than 52% of the genes expressed in brain.

**Table 2:** Genome Sizes and Predicted Protein Coding Gene Numbers from Insect Genomes

Organism	Genome Size Based on C-value	Gene Number
*Bombyx mori*	509-518 Mb	18,510
*Drosophila melanogaster*	117-176 Mb	13,854
*Anopheles gambiae*	264 Mb	13,683
*Apis mellifera*	166-342 Mb	10,157
*Tribolium castaneum*	196-205 Mb	9,132
*Danaus plexippus*	284 Mb	—

C-values are from Animal Genome Size Database [14]. The estimated gene numbers are from [50] for *B. mori*, [51] for *D. melanogaster*, [52] for *A. gambiae*, [53] for *A. mellifera,* and http://www.bioinformatics.ksu.edu/BeetleBase/index.shtml for *T. castaneum*.

Note: The Honeybee Genome Sequencing Consortium believes that their gene number is an underestimate.

### Functional annotation

Putative functional roles of the 6068 genes were analyzed by Gene Ontology (GO) annotation and compared to the *Drosophila* annotation (Table 3). The number of genes in the GO groups under Molecular Function and Biological Process was well represented in monarch EST resource. There were 148 genes grouped under behavior, which included 51 genes involved in learning and memory, 74 genes involved in locomotor activity, and 8 genes involved in visual behavior.

**Table 3:** Gene Ontology for Annotated Monarch Genes

Gene Ontology Terms	*Danaus Plexippus*	*Drosophila* Genome (FlyBase)
**Molecular function**	**4158**	**8849**
antioxidant activity (GO:0016209)	22	34
auxiliary transport protein activity (GO:0015457)	2	6
binding (GO:0005488)	2565	2474
catalytic activity (GO:0003824)	2120	3299
chaperone regulator activity (GO:0030188)	1	1
enzyme regulator activity (GO:0030234)	237	269
molecular transducer activity (GO:0060089)	371	515
motor activity (GO:0003774)	45	72
structural molecule activity (GO:0005198)	402	405
transcription regulator activity (GO:0030528)	419	609
transcription factor activity (GO:0003700)	147	300
translation regulator activity (GO:0045182)	72	83
transporter activity (GO:0005215)	545	650
**Biological process**	**4097**	**7393**
biological adhesion (GO:0022610)	216	152
biological regulation (GO:0065007)	1119	1455
cellular process (GO:0009987)	3626	4953
developmental process (GO:0032502)	1192	2244
establishment of localization (GO:0051234)	1026	732
growth (GO:0040007)	77	130
immune system process (GO:0002376)	108	197
localization (GO:0051179	1206	1074
locomotion (GO:0040011)	7	12
maintenance of localization (GO:0051235)	24	23
metabolic process (GO:0008152)	2816	3148
multicellular organismal process (GO:0032501)	1135	2366
multi-organism process (GO:0051704)	34	209
pigmentation (GO:0043473)	26	68
reproduction (GO:0000003)	373	698
reproductive process (GO:0022414)	86	203
response to stimulus (GO:0050896)	584	975
behavior (GO:0007610)	148	452
adult behavior (GO:0030534)	38	108
chemosensory behavior (GO:0007635)	37	169
feeding behavior (GO:0007631)	5	20
grooming behavior (GO:0007625)	4	10
hatching behavior (GO:0035187)	1	1
larval behavior (GO:0030537)	17	29
learning and/or memory (GO:0007611)	51	94
locomotory behavior (GO:0007626)	74	158
mechanosensory behavior (GO:0007638)	2	26
regulation of behavior (GO:0050795)	1	18
reproductive behavior (GO:0019098)	37	87
rhythmic behavior (GO:0007622)	24	34
visual behavior (GO:0007632)	8	11
rhythmic process (GO:0048511)	34	48

Number of assembled monarch (*D. plexippus*) sequences that were assigned into GO categories of Molecular function and Biological process based on BLASTX homology. The *Drosophila* number is provided as a reference.

### Using the EST database as a tool to investigate migration

Previous studies have focused on the physiological (e.g., reproductive diapause, increased longevity, cold tolerance, fat body hypertrophy) and behavioral (e.g., directional flight) aspects of monarch migration [2]–[4]. We are interested in expanding this knowledge to the molecular level, and the EST database is a powerful tool, as it will allow us to utilize microarray technology to identify candidate genes involved in all aspects of migration, with emphasis on those involved in migratory behavior.

As a prelude to microarray studies, we used a candidate gene approach, along with real-time polymerase chain reaction (qPCR), to evaluate potential differences in gene expression between summer and migratory butterflies using whole head homogenates. We examined the expression of four genes identified in the EST database that are involved in JH activity. The four genes are *allatotropin*, a neuropeptide that can stimulate JH synthesis in the corpora allata [15]; *juvenile hormone acid methyltransferase*, the enzyme that mediates the final step in JH biosynthesis [16]; *takeout*, a potential JH binding protein that is an output gene of the circadian clock and is implicated in feeding homeostasis [17]; and *juvenile hormone epoxide hydrolase*, an enzyme involved in JH degradation [18].

**Figure 2:**
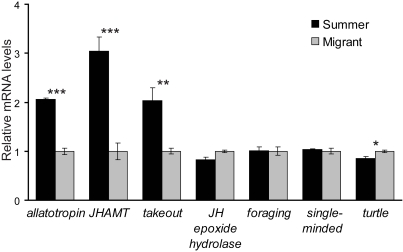
Expression profiles of selected genes between summer and migratory butterflies. Relative expression of the mRNA levels of *allatotropin*, *juvenile hormone acid methyltransferase* (*JHAMT*), *takeout*, *juvenile hormone (JH) epoxide hydrolase*, *foraging*, *single-minded*, and *turtle* were examined by qPCR. The analysis was performed on RNA from summer monarchs (three 12-animals sets of head RNA collected during summer 2005) and on RNA from migratory monarchs (three 12-animal sets of head RNA collected during fall 2005, and three 12-animals sets of head RNA collected during fall 2006). Only the 2005 RNA from migratory butterflies was used for analysis of *single-minded* and *turtle* gene expression. The results were normalized with *rp49* and then averaged. The average level of each gene in the migrants was normalized to 1.0 for graphing. *** p<0.001, ** p<0.01, * p<0.05

Consistent with increased JH activity in summer butterflies, *allatotropin*, *juvenile hormone acid methlyltransferase*, and *takeout* were each up regulated significantly in summer animals, compared to migrants *(allatotropin* and *juvenile hormone acid methyltransferase*, p<0.001; *takeout*, p<0.01) (Fig. 2). The levels of expression of the *juvenile hormone epoxide hydrolase* gene were not significantly different between migrants and summer monarchs (p>0.05), however. It has been reported that flight may help keep JH levels low during migration by enhancing JH degradation through the activity of JH esterase [19], which was not represented in our database.

We also examined the expression of the EST-identified monarch homologs for three genes involved in locomotor behavior, *foraging*, *single-minded*, and *turtle*. The *foraging* gene encodes a cyclic nucleotide-dependent protein kinase that was of particular interest because it has been shown to induce foraging behavior in bees [20], and some of the navigational activities of foraging bees resemble those of migratory monarchs (e.g., use of time-compensated sun compass orientation). The *single-minded* gene encodes a PAS-containing transcription factor involved in midline CNS development [21], and it is important for normal adult walking behavior and locomotion in flies [22]; *single-minded* mutant adult flies have defects in the central complex, which is an important integration center of visual and skylight information from eyes, and may be the actual site of the sun compass [23], [24]. The *turtle* gene encodes a CNS-specific member of the Ig superfamily that is required for coordinated motor control in *Drosophila* [25].

Interestingly the expression of *turtle* was significantly increased by 15% in migrants versus summer monarchs (p<0.05), making it a candidate gene involved in migratory locomotor behavior (Fig. 2). The expression of the *forager* and *single-minded* genes, however, were not significantly different between migrant and summer butterflies (p>0.05).

The results are consistent with the differential regulation of JH activity between summer and migratory butterflies and further suggest that *turtle* may be a candidate “migration” gene. However, the marginal increase in *turtle* expression in migrants needs to be re-examined in brains, as whole head extracts may not accurately reflect expression in brain. In addition, the brain distribution of expression of any candidate migration gene will need to be compared between migrant and summer butterflies.

### Circadian clock genes

The circadian clock in brain plays an important role in monarch migration by providing the timing component of time-compensated sun compass orientation [7]–[10], which contributes to successful navigation to the overwintering grounds. It is also possible that the circadian clock is involved in the induction of butterfly migration, as migration is initiated in the fall, in part, by decreasing daylength [26].

The EST database has allowed us to identify 8 monarch homologs out of the 12 genes involved in the core clock of *Drosophila* (Table 4). This included a *Drosophila*-like *cryptochrome*, designated insect *cry1*. Importantly, a novel, vertebrate-like *cryptochrome*, designated insect *cry2*, which is not present in *Drosophila,* was discovered in the monarch EST database [27]. This second *cry* encodes a light-insensitive protein that has potent repressive activity on the transcription factors CLOCK and CYCLE, which, as heterodimers, drive the intracellular transcriptional feedback loop that appears to be the critical gear of the molecular clock in all animals studied. The discovery of *cry2* has thus provided novel insights into the molecular nature of the monarch butterfly circadian clock in particular [28] and the diversity of insect clocks in general, as *cry2* exists in the genomes of all non-drosophilid insects so far examined [29].

**Table 4:** Clock Genes Represented in Monarch EST Database

Gene	EST Database	Proposed Function in *Drosophila* clock
*period*	—	Clock gene
*timeless*	—	Clock gene
*Clock*	—	Transcription factor
*cycle*	BF01012B2H07.f1	Transcription factor
*cryptochrome1*	BF14.3182.C1.Contig3165	Circadian photoreceptor
*cryptochrome2*	BF01037B1G10.f1BF01044A2E01.f1	N/A
*casein kinase II α*	BF14.2950.C1.Contig2954	Phosphorylates PERIOD
*casein kinase II β*	BF14.801.C1.Contig886	Forms tetramer with alpha subunit (α_2_β_2_)
*shaggy*	BF14.370.C1.Contig413*	Phosphorylates TIMELESS
*double-time*	BF01044A2C10.f1*	Phosphorylates PERIOD
*vrille*	BF14.1188.C1.Contig1279	Represses *Clk* transcription
*Pdp1ε*	BF01047A2E04.f1*	Activates *Clk* transcription
*slimb*	—	Ubiquitin-proteasome degradation of PERIOD

* 3′ UTR only.

For clock genes not found in the EST database, we cloned the complete open reading frames and 3′untranslated regions, which were then used to search the database using BLASTN. The proposed functions in *Drosophila* clock are from [54].

### A novel Na+/K+ ATPase allele for chemical defense

**Figure 3:**
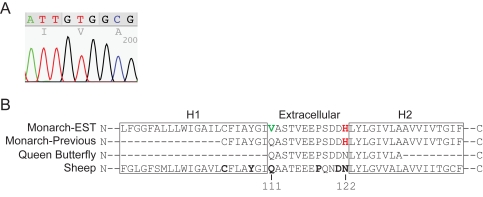
Na+/K+ ATPase in monarch butterflies. A. Electropherogram of valine codon in the BF01056B1B12.fl EST. B. Partial sequence of the Na+/K+ ATPase from the monarch, queen butterfly (*Danaus gilippus*), and sheep. H1 and H2 are transmembrane domains. The residues in bold in the sheep sequence have been shown by mutagenesis to confer ouabain resistance when mutated, and positions 111 and 122 are indicated [35]. The Monarch-EST sequence is from two EST clones (BF01056B1B12.fl & BF01017B2C11.fl) from the monarch EST database. The Monarch-Previous sequence is the previously reported monarch sequence which has an amino acid change at one critical site (shown in red) [32], [34]. Both of the EST sequences have a second amino acid change at a second critical site (shown in green). The queen butterfly sequence has neither change [34].

The utility of our EST resource for evaluating genes involved in the non-migratory aspects of monarch butterfly biology was apparent with the identification of ESTs encoding a new allele of a P type Na+/K+ ATPase (Fig. 3). The discovery of this novel allele bears directly on the chemical defense system of monarchs, as detailed below.

An intriguing aspect of monarch biology is the ability of the larvae to consume milkweed, which contains large amounts of cardiac glycosides. In most invertebrates and vertebrates, these compounds bind to and inhibit a ubiquitous P type Na+/K+ ATPase. Cardiac glycosides can cause death, because this sodium/potassium pump is essential for proper cardiac function. Monarchs store cardiac glycosides in their bodies through adulthood, and it acts as a chemical defense against predators [30], [31]. However, it has been shown that the monarch ATPase is resistant to inhibition by the cardiac glycoside, ouabain [32]. Furthermore, sequencing an extracellular domain involved in ouabain sensitivity revealed an amino acid change at a critical site (H122). Site-directed mutatgenesis of the naturally ouabain-sensitive *Drosophila* ATPase at this position (N122H) created a less sensitive enzyme [33]. Sequencing the extracellular domain from milkweed-feeding species closely related to monarch (i.e., the queen butterfly, *Danaus gilippus*) revealed that this amino-acid change was unique to *D. plexippus* [34].

We found two ESTs in the monarch database with high sequence similarity to this P type Na+/K+ ATPase. When these ESTs were translated and aligned with the previously reported monarch sequence, an additional amino acid change was identified within this ouabain-sensitive domain (Fig. 3). This change is a result of not one but two nucleotide transversions; the CAG codon encoding glutamine is replaced by the GTG codon encoding valine (CA→GT). Interestingly, this particular position (amino acid 111) also has been shown to be important for ouabain sensitivity; amino acid substitutions produced by a random mutagenesis in the sheep α1 Na+/K+ ATPase at this site conferred ouabain resistance (Q111L, Q111R, Q111H) [35]. Lastly, when both position 111 and 122 were mutated in the same clone, ouabain resistance was higher than when a single mutation was present [36]. It is quite likely that the Na+/K+ ATPase variant present in the EST database is more resistant to ouabain than the allele previously reported.

### Single nucleotide polymorphisms and microsatellites as genetic markers

The identification of single nucleotide polymorphisms (SNPs) and microsatellite polymorphisms will be useful for population studies of monarch butterflies at the species and subspecies levels. As nearly 300 individual wild butterflies collected from three states (Massachusetts, Minnesota, and Texas) were used to construct the EST library (Table 1), high polymorphism levels are expected to be present within the library. We took advantage of this expectation to identify SNPs and polymorphisms between microsatellite sequences.

To find reliable SNPs, we used a “double-hit” criterion in which each allelic variant must be represented by two or more ESTs (see Methods). Indeed, 1599 double-hit SNPs were identified from the 3,486 contigs (Dataset S2). To find microsatellites, we searched for tandem repeat sequences of 2, 3, 4, and 5 nucleotide repeats within our EST database. We identified 1333 potential microsatellites, and 98 of these exhibited polymorphism (Table 5, Dataset S2).

**Table 5:** Microsatellites Found in Monarch ESTs

Repeat Size	Repeat #	Total	Polymorphic*
2	>5	511	61
3	>3	739	29
4	>3	64	3
5	>3	19	5

* Number of microsatellites that exhibit polymorphism.

These SNPs and microsatellite polymorphisms can be used to more extensively address the long-standing question of the population structure of North American monarchs. Tagging studies have shown that monarchs from the Eastern United States of America (USA) overwinter in Mexico, while monarchs from the Western USA (west of the Rocky Mountains) overwinter in California [37]. Thus, it has been hypothesized that the Eastern and Western monarchs are two geographically isolated populations. Prior genetic studies using mtDNA [38], [39] have shown that Eastern and Western (and non-migrating South American) monarchs are rather homogenous with no clear population structure. [Also, Eanes and Koehn [40] found little variation in allozyme alleles within Eastern monarchs].

In addition to the issues of population structure, the SNPs and microsatellite polymorphisms found in our EST database will be useful for analyzing genetic differences between naturally occurring migrating (North American) and non-migrating (South American) subspecies [41], [42]. Furthermore, the SNP data could be used to identify genes that are evolving under natural selection (e.g., [43]).

### Conclusions

To our knowledge, the monarch brain EST resource provides the first analysis of a brain transcriptome for any butterfly species. Our results show that the EST database will be valuable for examining the molecular control of many aspects of monarch butterfly biology. Likewise, the results suggest that extensive, unbiased analysis of differential gene expression between summer and migratory butterflies using high-density microarrays of all 9484 unique sequences will be informative for uncovering the genes involved in migratory behaviors. The SNPs and microsatellite polymorphisms offer important genetic markers for more rigorous analysis of North American monarch population structure and subspecies differences between migrating and non-migrating monarchs, than has been possible previously. Our monarch EST resource adds significantly to the expanding, comparative genomic data already available in Lepidoptera [44]. The resource also sets the stage for the cloning of the monarch butterfly genome.

## Materials and Methods

### Monarchs used for cDNA library

A total of 298 monarch butterfly brains were collected to construct the cDNA library (Table 1). Mid-summer, late-summer, and fall butterflies were obtained to ensure transcripts from both reproductive and diapuasing/migratory animals were represented in the library. Mid-summer butterflies were caught between August 11–14, 2004, near Greenfield, Massachusetts, USA (latitude 42°59′N, longitude 72°60′W) by Fred Gagnon, late-summer butterflies were caught between September 5–7, 2004, near Cannon Falls, Minnesota, USA (latitude 44°52′N, longitude 92°90′W) by Tim Murphy, and migrating butterflies were collected from roosts between October 19–10, 2004 near Eagle Pass, Texas, USA (latitude 28°71′N, longitude 100°49′W) by Carol Cullar. Mid-summer butterflies were housed in cages outside, and late-summer and fall butterflies were housed in glassine envelopes in incubators with controlled temperature (18°C), humidity (70%), and lighting (which mimicked the prevailing outdoor light-dark conditions) for less than one week prior to brain collections. The butterflies were fed 15% sucrose every other day.

Brains were collected in both the morning and the afternoon to increase chances of including circadian-controlled transcripts. Fresh brains were dissected in 0.5× RNAlater (Ambion). Brains did not include the photoreceptor layer of the eye.

To confirm that the Texas butterflies were in diapause, the female abdomens were dissected to determine reproductive status; none contained mature oocytes.

### cDNA library construction, sequencing, and analysis

The W. M. Keck Center for Comparative and Functional Genomics (University of Illinois at Urbana-Champaign) carried out the following using the protocol of [45]:

Total RNA was extracted from each group of brains above using Trizol (Invitrogen), and equal amounts of RNA from mid-summer, late-summer, and fall (migratory) butterflies were pooled. PolyA+ RNA was purified from the total RNA mix using the Oligotex Direct mRNA kit (Qiagen). The mRNA was reverse transcribed using a polydT primer with a tag sequence appended. Double-stranded cDNAs larger than 800 bp were directionally cloned into a NotI and EcoRI digested pBS II SK(+) vector (Stratagene). After normalizing the primary library, 10,176 clones were sequenced to a redundancy of 41%. The average insert size of 12 clones was 1.7 kb (based on PCR of inserts). This library was then subtracted, and another 11,063 clones were sequenced.

The 5′ ends of the inserts were sequenced with a single pass. Sequences with a length of more than 200 base pairs after the quality trimming process were considered “high-quality”, while sequences that failed at this stage were called “low quality”. Next, the vector sequence was removed. If the remaining sequence length was less than 200 base pairs, then the sequence was called “short insert” and was removed from further analysis. Lastly, sequences were “filtered” for possible contaminants such as the *E. coli* genome, vector DNA, mitochondrial DNA, ribosomal RNA, and viral DNA using BLASTN. The remaining sequences were the “clean” sequence set. The raw sequences from the “clean” set (available in Dataset S1) were assembled into contigs using *Phrap*, and the vector sequences were trimmed from the contigs. All contigs were inspected manually using *Consed,* and a non-redundant database search detected false contigs.

### Differential gene expression studies between summer and migratory butterflies

Summer butterflies were reared outdoors in western Massachusetts by Fred Gagnon. Adults were held in cages outside until mating was observed, which is indicative of mature reproductive status. On September 1, 2005 whole heads from 36 butterflies were collected and divided into three 12-animal sets for total RNA analysis.

Migrating butterflies were caught in Texas by Carol Cullar (October 17, 2005; October 16, 2006) and housed in an incubator for one week at 18°C prior to head collections. To confirm diapause status, 10 female abdomens were dissected and no mature oocytes were found. In addition, five male abdomens were dissected, and ejaculatory duct/tubular gland wet weights were less than 16 mg. Overwintering males have low reproductive organ weights [46], while males housed in summer conditions (25°C, 16 hrs light per day) have ejaculatory duct/tubular gland wet weights that average 32.4 mg [47]. Whole heads were collected from 36 of the 2005 migrants and 36 of the 2006 group; each of the two groups was divided into three 12-animal sets for RNA analysis.

Total RNA was prepared from each set of summer or migrating heads using Trizol (Invitrogen), and pigments were removed from the total RNA using charcoal purification.

Real-time PCR was performed using Taqman PCR primer/probe sets, and *rp49* was used as control. For each candidate gene, the EST used for primer design was: *allatotropin*, BF01058B2A04.f1; *juvenile hormone acid methyltransferase,* BF01030B2G02.f1; *takeout*, BF01062B1H01.f1; *juvenile hormone epoxide hydrolase*, BF01057A1H08.f1; *foraging*, BF01042B1A03.f1; *single-minded*, BF01007X1C02.f1; and *turtle*, BF01052B1D11.f1. The primers and probe for *rp49* were described previously [7]. The other primers and probes were as follows (F = forward primer, R = reverse primer, P = probe, all 5′-3′); *allatotropinF*: CCCGAGGGTTGGTAAACTTCA, *allatotropinR* : GGCTCGTGTTGCTCAATCCT, *allatotropinP*: FAM- AGCCCGTAGCTTTGGAAAACGCGA-BHQ1; *juvenile hormone acid methyltransferaseF*: GAACATCACGCCATGGATAACA, *juvenile hormone acid methyltransferaseR*: CGAAGTTCATCAGGCAGTTCAC, *juvenile hormone acid methyltransferaseP*: FAM-CAGCTTCACGCGGCTCGACATAGA-TAMRA; *takeoutF*: TCAGAACCAGTGCTACATTTTAAGGA, *takeoutR*: TGTTGTATCCATTTTAAACCCAGAAA, *takeoutP*: FAM-CTAACGGTTACAGGATTGAAGGGTCA-BHQ1; *juvenile hormone epoxide hydrolaseF*: ATGATTTAAGGGAGAGGTTGCTACA, *juvenile hormone epoxide hydrolaseR*: AACCGTAAGTGAAGCCTGAATTTTC, *juvenile hormone epoxide hydrolaseP*: FAM-TCGGCCATTTCAGCCTCCTC-BHQ1; *foragingF*: CCTTCAACCAGCTTATCTC, *foragingR*: TCATCGCCAACATCCT, *foragingP*: FAM- ACGCTCGATGAAATCCGCACCA-BHQ1; *single-mindedF*: GCCGTCACCGAGCTGAAG, *single-mindedR*: TGGCGTCCAGGAAGATGAG, *single-mindedP:* FAM-ATGTTCATGTTCCGCGCCTCGC-TAMRA; and *turtleF*: GGGTCAAACACAAGGCCATAAC, *turtleR*: ACGGACAGTATGATGGCCACTA, *turtleP*: FAM-TCGTTGGAGGGATATTGTTCTTC-TAMRA.

### SNP and microsatellite identification

To identify SNPs in the EST database, trimmed EST sequences were assembled into contigs using *Phrap* developed by Phil Green (University of Washington) (http://www.phrap.org/). SNPs were predicted using the SEAN program (http://zebrafish.doc.ic.ac.uk/Sean/) [48]. To reduce the number of false SNPs due to sequencing or reverse transcription errors, the search for SNPs was restricted to contig regions with at least four-fold coverage, and a SNP was defined as a base variation that is present in at least two EST sequences. To remove sequences with potential sequencing errors, 15 base pairs on either side of the polymorphic position were compared to the consensus; if a second polymorphism was detected, this sequence read was eliminated from the analysis.

Microsatellite repeats were identified using a custom PERL script [49] on *Phrap* assembled contigs and singlet sequences. The location and size of each microsatellite is listed in the supplemental material. Default cutoffs (more than 5 repeats for 2bp, more than 3 repeats for 3bp, 4bp, and 5bp) were used for positive identification. Polymorphisms were detected by visual inspection of all microsatellites using a contig viewer program (sean.jar) provided in the SEAN program package. Summaries and details for both SNPs and microsatellites are provided in Supporting Dataset S2.

## Supporting Information

Dataset S1 Monarch Butterfly ESTs. A compressed FASTA file contains all the EST sequences in the monarch database.
(4.67 MB ZIP)

Dataset S2 SNPs and Microsatellites in the Monarch EST Database. A compressed file contains detailed information of SNPs and mcirosatellites in the monarch EST database, which includes summaries for both SNPs and microsatellites. Access to the sequence information and position for each SNP and microsatellite is also provided.
(17.63 MB ZIP)
